# Supplementary material for: Shared Treg dysregulation underlies psoriasis vulgaris and atopic dermatitis through the PHF19-PRC2 epigenetic axis
Source: Front Immunol. 2026 Aug 20;17:1932788. doi: 10.3389/fimmu.2026.1932788 (PMC13538097; doi:10.3389/fimmu.2026.1932788)
Supplement: Supplementary file 3 [file DataSheet3.zip › Additional files/Additional file 5/64.SRA1.scatter.pdf]

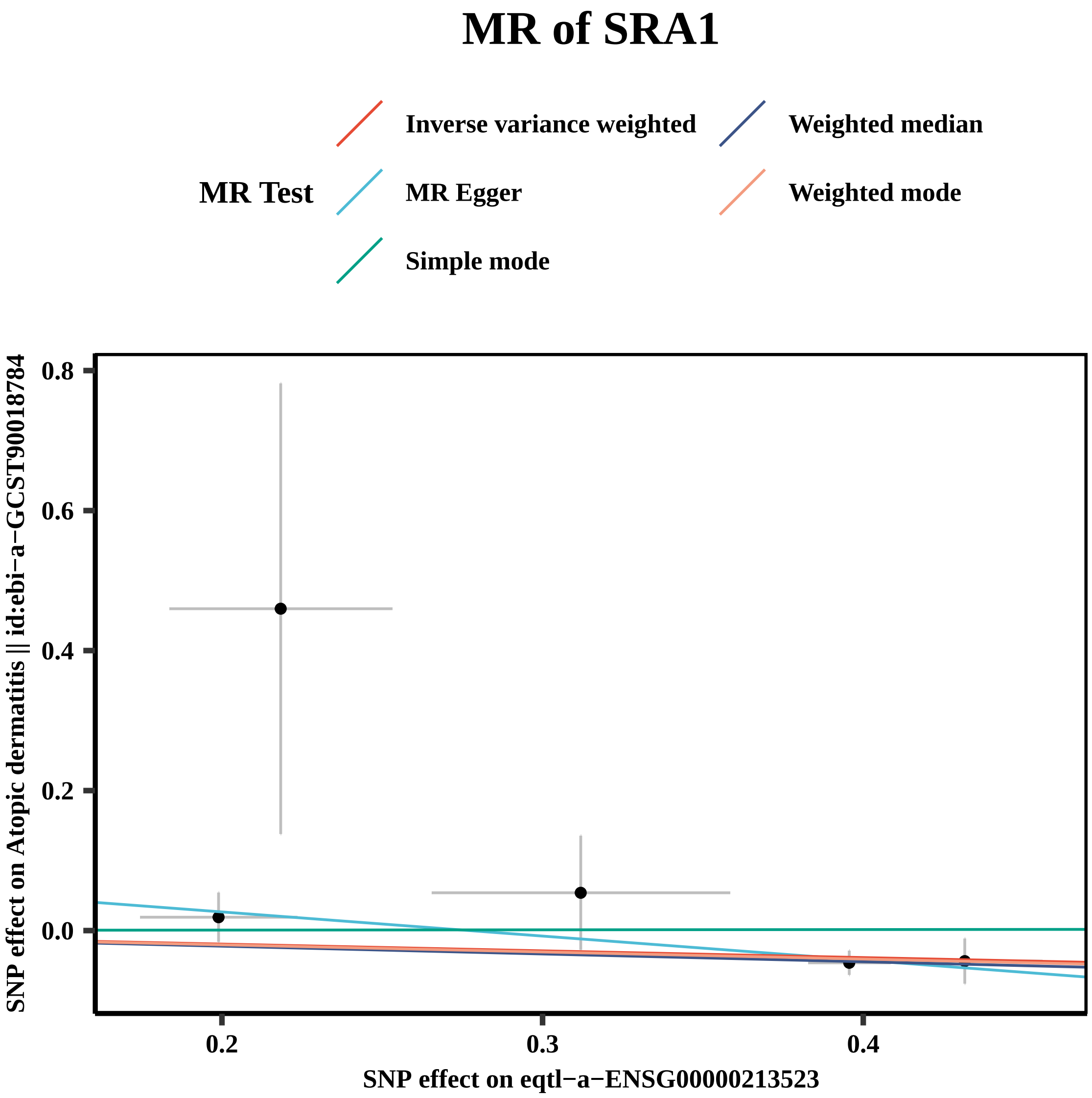
MR of SRA1
MR Test
Inverse variance weighted
MR Egger
Simple mode
Weighted median
Weighted mode
0.8
0.6
0.4
0.2
0.0
SNP effect on Atopic dermatitis || id:ebi−a−GCST90018784
0.2
0.3
0.4
SNP effect on eqtl−a−ENSG00000213523
